# Access to and use of long-lasting insecticidal nets and factors associated with non-use among communities in malaria-endemic areas of Al Hudaydah governorate in the Tihama region, west of Yemen

**DOI:** 10.1186/s12936-017-1894-9

**Published:** 2017-06-09

**Authors:** Samira M. A. Al-Eryani, Mohammed A. K. Mahdy, Abdulsalam M. Al-Mekhlafi, Rashad Abdul-Ghani

**Affiliations:** 1 Department of Parasitology, Faculty of Medicine & Health Sciences, Sana’a University, Sana’a, Yemen; 2 Tropical Disease Research Center, Faculty of Medicine and Health Sciences, University of Science and Technology, Sana’a, Yemen

**Keywords:** Long-lasting insecticidal net, LLIN ownership, LLIN access, LLIN use, Al Hudaydah, Tihama

## Abstract

**Background:**

Universal coverage of the targeted malaria-endemic areas with long-lasting insecticidal nets (LLINs) is implemented as one of the key interventions for malaria control and elimination in Yemen. In 2013, through a mass campaign, LLINs were distributed to the targeted communities in Al Hudaydah governorate. This study aimed to assess the ownership of, access to, and use of LLINs. It also aimed to identify factors associated with not using LLINs in malaria-endemic areas of Al Hudaydah in the Tihama region, west of Yemen.

**Methods:**

A cross-sectional survey was conducted in four districts (Ad Durayhimi, Al Marawi’ah, Al Mansuriyah and Bayt Al Faqiah) in Al Hudaydah during February 2016. A total of 701 households were included in this study. Data on socio-demographic characteristics and availability of LLINs were collected by interview and observation. Four indicators for malaria prevention using LLINs; proportion of households with at least one LLIN, proportion of households with at least one LLIN for every two people, proportion of population with access to LLINs in the surveyed households and proportion of population who slept under LLINs the previous night of the survey were calculated as indicated by Roll Back Malaria Monitoring and Evaluation Reference Group. Use to access ratio was assessed. Factors associated with not using LLINs among people with access were also investigated.

**Results:**

Of 701 households with 4900 de facto population, ownership of at least one LLIN was 90.6%, while 24.1% owned at least one for every two people during the survey in 2016. The overall proportion of people with access to LLINs was 51.5% (95% CI 50.1–52.9). Only 19.0% (95% CI 17.9–20.1) slept under LLINs the night before the survey and the overall use to access ratio was 0.37. The proportions of children under 5 years of age with access to and use of LLINs were 13.7 and 42.5%, respectively. On the other hand, the proportions of pregnant women with access to and use of LLINs were 16.4 and 20.0%, respectively. Multivariable analysis identified that people living in Al Mansuriyah district [adjusted odds ratio (AOR) = 3.29, 95% confidence interval (CI)  1.35–8.01; *P* = 0.009)], having three or more damaged LLINs in the house (AOR = 2.76, 95% CI 1.79–4.25; *P* < 0.001), aged between 16 and 45 years old (AOR = 2.17, 95% CI 1.26–3.75; *P* = 0.005) or older (AOR = 2.17, 95% CI 1.09–4.29; *P* = 0.026) and living in huts (AOR = 1.59, 95% CI 1.09–2.32; *P* = 0.015) were significantly less likely to use LLINs.

**Conclusions:**

This study shows a low LLIN access rate among local communities targeted for universal LLIN coverage in Al Hudaydah, a malaria-endemic area of high transmission. This finding necessitates additional distribution channels following mass campaigns to maintain the universal coverage. Reduced use of LLINs among people with access in these communities together with the identified risks of non-use highlight the importance of conducting behaviour change communication campaigns to enhance using LLINs in areas with universal coverage.

## Background

Yemen is one of the six countries in the Eastern Mediterranean region that continues to have areas of high malaria transmission and contributes to the majority of malaria cases in the Arabian Peninsula [1, 2]. A regional strategy with the ‘malaria-free Arabian Peninsula’ initiative by the year 2020 was proposed by the Eastern Mediterranean Regional Office of the World Health Organization (WHO, EMRO) in 2004 to maintain the malaria-free status in the Gulf states and support malaria elimination from Yemen [2, 3]. The current updated Yemeni National Malaria Control and Elimination Strategy (NMCES) for 2014–2018 supports this initiative with the overall aim to eliminate the disease from the country by the year 2020 and includes collaboration with the Gulf Cooperation Council countries for funding, specifically the coordinated Saudi-Yemeni cross-border vector control and surveillance activities [2, 4].

Two mainstream vector control intervention tools, indoor residual spraying (IRS) and insecticide-treated nets (ITNs) in the form of long-lasting insecticidal nets (LLINs), form the vector control component of the NMCES (2014–2018) [4]. Based on the present malaria stratification in Yemen, universal coverage with LLINs, alone or in combination with IRS, is implemented in three altitude-based epidemiological strata; stratum 1 (0–600 m above sea level), stratum 2 (601–1000 m above sea level) and stratum 3 (1001–1500 m above sea level) [4]. In Yemen, LLINs were first introduced in 2006 [5, 6], and prior to 2011, their distribution only targeted the vulnerable population groups, children under 5 years of age and pregnant women. Distribution of LLINs is free of charge to all ages through mass distribution campaigns, which is currently the only distribution channel for this intervention in Yemen [4]. One LLIN for every two people, as recommended by the WHO [7], is distributed with the aim of protecting rural populations within the targeted malaria-endemic areas at altitudes between 0 and 1500 m above sea level, which include the highest burden governorates (Al Hudaydah and Hajjah) [4].

The *‘proportion of households owning at least one ITN*’ and ‘*proportion of children under 5* *years or pregnant women who slept under an ITN the previous night*’ were the previously recommended two principal indicators to measure the ownership and use of ITN as a malaria prevention tool [8]. However, these indicators are limited by not identifying if actual use was due to inadequate ITNs within a household or due to behavioural factors [9, 10]. Eisele et al. [9] reported that only by achieving intra-household universal access of ‘*two people per ITN’* can surveys interpret actual use among children under 5 years and pregnant women, following which behaviour change communication (BCC) programmes can then reduce the gap between ITN use among these vulnerable populations within households with access to ITNs.

Following revision of the indicators by the ‘Survey and Indicator Guidance Task Force’ of the RBM Monitoring and Evaluation Reference Group (MERG) in 2011, additional ‘‘*new core indicators were proposed: the proportion of households with at least one ITN for every two people and the proportion of population that had access to ITN within the household*” [10, 11]. Two malaria indicator surveys (MIS) were conducted in Yemen during 2009 and 2013, both of which did not include assessing the proportion of population that had access to LLIN within the household and the use among the population with access [12, 13]. Therefore, this study assessed the universal LLIN coverage by applying the indicators approach developed and recommended by the MERG and identified the factors associated with not using LLINs among people with access to LLINs (one LLIN for every two people) in universally covered malaria-endemic areas of Al Hudaydah in the Tihama region of Yemen.

## Methods

### Study design and setting

This cross-sectional study was conducted in rural malaria-endemic areas of Al Hudaydah during February 2016, in the transmission season when using LLINs is expected to be at the highest level. Al Hudaydah is located in the western coastal plain of Yemen at the coordinates of 14°48′08″N 42°57′04″E, bordering the Red Sea with a total area of 17,509 km^2^. As per the latest census, it has a total population of 2,279,000 [14]. The temperatures vary from 27 to 42 °C and low to very low rainfall (<200 mm/year) [15]. The rains usually occur during February to April and September to October. The transmission season of malaria lasts for about 6 months from November to April, and *Anopheles arabiensis* has been incriminated as the principal malaria vector [16, 17]. Al Hudaydah is one of the highest malaria burden and transmission governorates in the country and, therefore both IRS and LLINs are implemented in the targeted malaria areas for the prevention and control of malaria [4]. In 2013, a mass campaign was conducted to distribute LLINs to the targeted malaria-endemic communities in Al Hudaydah. Two brands, Yorkool^®^ (Tianjin Yorkool International Trading Co., Ltd.), and NetProtect^®^ (Intelligent Insect Control, Bestnet A/S), which are rectangular and measuring 190 × 180 × 150 cm, were distributed.

### Sampling strategy

**Fig. 1:**
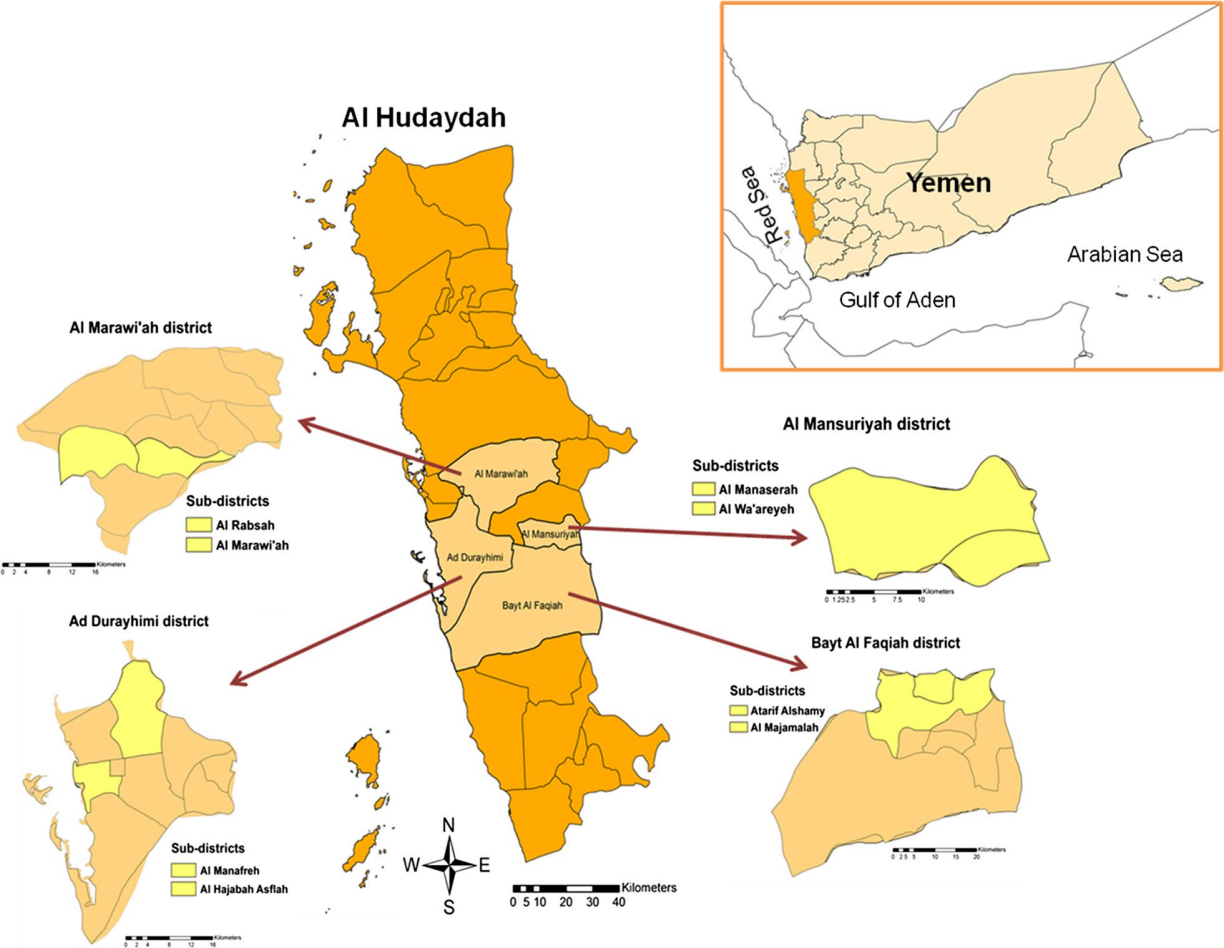
Map for the study area in Al Hudaydah governorate, in the Tihama region of Yemen

A multi-stage sampling strategy was adopted. Briefly, four districts (Ad Durayhimi, Al Marawi’ah, Al Mansuriyah and Bayt Al Faqiah) were randomly selected from a list of the districts covered with LLINs during the distribution campaign implemented by the National Malaria Control Programme (NMCP) in 2013. Within each district, two sub-districts were then randomly selected, and from which households were sampled by random sampling. The number of households sampled from each district and sub-district was proportional to the population size of the district/sub-district (Fig. 1). Sample size was calculated by Epi Info™ 7.1.3 (CDC, Atlanta, USA), assuming an outcome frequency of 50.0%, a 95% confidence level and an estimated design effect of 1.5. Using these criteria, a minimum sample size of 575 households receiving LLINs was required. An additional 20% of households were added to the sample size to allow for non-response, unusable data or other limitations.

### Data collection

Data were collected using well-structured questionnaires, adapted from research tools previously used in the project ‘Evaluation of Bed Nets in Loreto, Peru’ (V. Paz Soldan and A. Lenhart, pers. comm., 2015), which were designed in English and translated into Arabic. The questionnaires included biodata, socioeconomic data, housing status and observation of the available LLINs with all household members. The data was collected by interview with the head of household or, if not available, an adult member of the household. The LLIN brand and the presence and size of holes were recorded. The WHO hole size which is classified into 4 sizes was used; “*size 1: smaller than a thumb, size 2: larger than a thumb but smaller than a fist, size 3: larger than a fist but smaller than a head and size 4: larger than a head*” [18]. If small holes (size 1) were only present on one side, the LLIN was not considered damaged. Damaged nets were defined as nets with size-1 or/and size-2 holes being observed on more than one side of the net, or the presence of size-3 or size-4 holes.

### Data analysis

Survey data were analysed using the IBM SSPS Statistics for Windows, version 22.0 (IBM Corp., Armonk, NY, USA). Four main indicators for the assessment of malaria prevention using LLINs; proportion of households with at least one LLIN, proportion of households with at least one LLIN for every two people, proportion of population with access to LLINs in the surveyed households and proportion of population who slept under LLINs the previous night were calculated as recommended by the RBM MERG [11, 19], where the first and the second parameters were calculated from the total number of the households while the third and the fourth parameters were calculated from the de facto population (slept in the house at the previous night). The indicator for proportion of population with access gives an estimate of the proportion of population that could have slept under a LLIN (assuming each LLIN can be used by two people). Therefore, an intermediate variable of “potential users” was first created by multiplying the number of LLINs in each household by a factor of 2.0. The potential users were modified to equal the de facto population in the household if they were more than the number of the people in the household. Then, the indicator was calculated by dividing the sum of all potential users in the sample by the de facto population [19]. The 95% confidence interval (CI) for each proportion was calculated. In addition, the use to access ratio was calculated by dividing the result from the ‘use’ indicator by the result from the ‘access’ indicator [20]. For the risk assessment of not using LLINs, the dependent variable was identified as not using a LLIN among household members with universal coverage to LLINs (one LLIN for every two people) the night prior to the survey. The independent variables included age, gender, number of women of childbearing age (15–49 years), presence of pregnant women, family size, house structure, districts, socio-economic status (SES), and number of damaged LLINs within a household. The SES was estimated based on the principal component analysis (PCA) of durables owned by households. Therefore, the constructed PCA-based scores of households were included in the statistical analysis as continuous independent variables and divided into quintiles. According to this approach, the lowest 40%, the middle 20% and the highest 40% of the households were classified as being of low, middle and high SES, respectively [21]. The association between independent and dependent variables was tested using Pearson’s Chi square test, with reporting the corresponding odds ratio (OR) and its 95% CI. Multivariable analysis using a conditional forward stepwise logistic regression model was applied to all variables in the bivariate analysis, and the adjusted OR with its 95% CI was also reported.

## Results

### Characteristics of study subjects

Of the de facto population surveyed, 50.7% (2486/4900) were males. The median age of the population was 17 years (interquartile range of 21 years). The main household durables owned by households included a television (32.3%), a stereo system or radio (26.3%) and a motorcycle (24.9%). Fewer households owned a washing machine (7.4%), a gas stove (6.2%), a vehicle (5.3%), a bicycle (3.9%), a refrigerator (3.0%) or an electric generator (1.5%). Houses in the study area were typically structured as single or attached rooms, or as huts with thatch roofs. A few houses were two floor structures.

### Characteristics of LLINs

A total of 1348 LLINs were observed among the households in the study areas during the survey. Of the nets with retained labels (n = 1082), the brands of the observed LLINs were Yorkool^®^ (Tianjin Yorkool International Trading Co., Ltd.), NetProtect^®^ (Intelligent Insect Control, Bestnet A/S), PermaNet^®^ 2.0 (Vestergaard Frandsen), Royal Sentry^®^ (Disease Control Technologies LLC) and Olyset^®^ (Sumitomo Chemical Company, Ltd.). The majority of the LLINs observed were of the brands Yorkool^®^ (61.0%; 660/1082) and NetProtect^®^ (34.2%; 370/1082), which were distributed during the mass campaign in 2013. All LLINs were rectangular, and the colour of the majority was green/dark green.

### Ownership, access to and use of LLINs

The overall proportion of households that owned at least one LLIN was 90.6% (635/701), while 24.1% (169/701) of the households owned at least one LLIN for every two household members. Among the districts, the highest proportion of households owning at least one LLIN was observed in Al Durayhimi (96.1%; 95% CI 88.1–99.0), while the lowest proportion was observed in Al Marawi’ah (89.2%; 95% CI 83.1–93.4). On the other hand, the highest proportion of households owning at least one LLIN for every two people was observed in Al Mansuriyah (27.8%; 95% CI 16.9–41.9), while the lowest proportion was observed in Al Marawi’ah (13.3%; 95% CI 8.6–19.8) (Table 1).

**Table 1:** Ownership of LLINs by local communities of Al Hudaydah governorate in 2016

Surveyed area	Number of households	Households with at least one LLIN % (95% CI)	Households with at least one LLIN for every two people % (95% CI)
Overall	701	90.6 (88.1–92.6)	24.1 (21.0–27.5)
Districts/Sub-districts			
Ad Durayhimi	76	96.1 (88.1–99.0)	27.6 (18.3–39.3)
Al Manafreh	39	97.4 (84.9–99.9)	15.4 (6.4–31.2)
Al Hajabah Asflah	37	94.6 (80.5–99.1)	40.5 (25.2–57.8)
Al Marawi’ah	158	89.2 (83.1–93.4)	13.3 (8.6–19.8)
Al Marawi’ah	106	86.8 (78.5–92.3)	12.3 (7.0–20.4)
Al Rabsah	52	94.2 (83.1–98.5)	15.4 (7.3–28.6)
Al Mansuriyah	54	90.7 (78.9–96.5)	27.8 (16.9–41.9)
Al Manaserah	30	86.7 (68.4–95.6)	20.0 (8.4–39.1)
Al Wa’areyeh	24	95.8 (76.9–99.8)	37.5 (19.6–59.2)
Bayt Al Faqiah	413	90.1 (86.7–92.7)	27.1 (22.9–31.7)
Al Majamalah	135	89.6 (82.9–94.0)	35.6 (27.7–44.3)
Altarif Alshamy	278	90.3 (86.0–93.4)	23.0 (18.3–28.5)

The overall proportion of the surveyed population with access to LLINs was 51.5% (95% CI 50.1–52.9), where it was the highest in Ad Durayhimi (58.6%; 95% CI 54.0–63.0) and the lowest in Al Marawi’ah (42.9%; 95% CI 40.1–45.7). The overall proportion of population that used LLINs the previous night was 19.0% (95% CI 17.9–20.1), with the highest LLIN use in Bayt Al Faqiah (22.1%; 95% CI 20.6–23.7). However, the overall ratio of use to access was 0.37 (Table 2). Among the vulnerable populations, the proportions of children under 5 years with access to and use of LLINs were 13.7% (95% CI 11.1–16.8) and 42.5% (95% CI 31.7–54.1), respectively. However, the proportions of pregnant women with access to and use of LLINs were 16.4% (95% CI 8.6–28.5) and 20.0% (95% CI 3.5–55.8), respectively.

**Table 2:** Access to and use of LLINs by local communities of Al Hudaydah governorate in 2016

Surveyed area	Population (de facto)	Population that used LLINs the night prior to the survey^a^ % (95% CI)	Population with access to LLINs within their household % (95% CI)	Ratio of use to access
Overall	4900	19.0 (17.9–20.1)	51.5 (50.1–52.9)	0.37
Districts/Sub-districts				
Ad Durayhimi	483	19.7 (16.3–23.6)	58.6 (54.0–63.0)	0.34
Al Manafreh	253	9.1 (6.0–13.5)	56.5 (50.2–62.7)	0.16
Al Hajabah Asflah	230	31.3 (25.5–37.8)	60.9 (54.2–67.2)	0.51
Al Marawi’ah	1204	13.4 (11.5–15.5)	42.9 (40.1–45.7)	0.31
Al Marawi’ah	850	13.4 (11.2–15.9)	40.9 (37.6–44.3)	0.33
Al Rabsah	354	13.3 (10.0–17.4)	47.5 (42.2–52.8)	0.28
Al Mansuriyah	351	12.3 (9.1–16.3)	53.3 (47.9–58.6)	0.23
Al Manaserah	195	13.9 (9.5–19.7)	50.3 (43.1–57.5)	0.28
Al Wa’areyeh	156	10.3 (6.2–16.4)	57.1 (48.9–64.9)	0.18
Bayt Al Faqiah	2862	22.1 (20.6–23.7)	53.7 (51.9–55.5)	0.41
Al Majamalah	881	12.7 (10.6–15.1)	59.4 (56.0–62.6)	0.21
Altarif Alshamy	1981	26.3 (24.3–28.3)	51.2 (49.0–53.4)	0.51

^a^Calculated for households with at least one LLIN

### Factors associated with the non-use of LLINs

Bivariate analysis showed that residents of Al Mansuriyah (OR = 4.68, 95% CI 2.03–10.83; *P* < 0.001), having three or more damaged nets per household (OR = 3.10, 95% CI 2.10–4.56; *P* < 0.001), being poor (OR = 2.03, 95% CI 1.35–3.07; *P* = 0.001), household members of age groups 16–45 (OR = 1.92, 95% CI 1.17–3.15; *P* = 0.010) or older (OR = 1.86, 95% CI 1.02–3.39; *P* = 0.043) and living in huts (OR = 1.62, 95% CI 1.16–2.26; *P* = 0.004) were the factors significantly associated with not using LLINs the night preceding the survey. The multivariable analysis confirmed that residents of Al Mansuriyah (AOR = 3.29, 95% CI 1.35–8.01; *P* = 0.009), having three or more damaged nets per household (AOR = 2.76, 95% CI 1.79–4.25; *P* < 0.001), age groups of 16–45 years (AOR = 2.17, 95% CI 1.26–3.75; *P* = 0.005) or older (AOR = 2.17, 95% CI 1.09–4.29; *P* = 0.026) and living in huts (AOR = 1.59, 95% CI 1.09–2.32; *P* = 0.015) are independent risk factors for not using LLINs (Table 3).

**Table 3:** Factors associated with not using LLINs the night prior to the survey in Al Hudaydah governorate in 2016

Characteristics	Population with access (de facto)^a^	People that did not sleep under LLINs n (%)	OR (95% CI)	AOR (95% CI)	*P* value
Age (years)					
<5	80	46 (57.5)	Reference	Reference	
5–15	220	145 (65.9)	1.43 (0.85–2.41)	1.34 (0.76–2.36)	0.307
16–45	377	272 (72.1)	1.92 (1.17–3.15)	2.17 (1.26–3.75)	0.005*
>45	116	83 (71.6)	1.86 (1.02–3.39)	2.17 (1.09–4.29)	0.026*
Presence of pregnant women					
Yes	10	8 (80.0)	Reference		
No	783	538 (68.7)	0.55 (0.12–2.60)	0.70 (0.13–3.72)	0.670
Number of women of child bearing age (15–49 years)					
0	74	51 (68.9)	Reference	Reference	
1	435	279 (64.1)	0.81 (0.48–1.37)	0.88 (0.49–1.59)	0.674
≥2	284	216 (76.1)	1.43 (0.82–2.52)	1.25 (0.66–2.35)	0.488
Gender					
Female	395	275 (69.6)	Reference	Reference	
Male	398	271 (68.1)	0.93 (0.69–1.26)	1.05 (0.76–1.45)	0.785
Family size					
≤5	346	230 (66.5)	Reference	Reference	
>5	447	316 (70.7)	1.22 (0.90–1.65)	1.04 (0.72–1.52)	0.823
House structure					
Not hut	528	346 (65.5)	Reference	Reference	
Hut	265	200 (75.5)	1.62 (1.16–2.26)	1.59 (1.09–2.32)	0.015*
Number of damaged LLINs within a household					
0	293	174 (59.4)	Reference	Reference	
1	74	44 (59.5)	1.00 (0.60–1.69)	0.98 (0.56–1.71)	0.946
2	155	106 (68.4)	1.48 (0.98–2.23)	1.46 (0.94–2.27)	0.090
≥3	271	222 (81.9)	3.10 (2.10–4.56)	2.76 (1.79–4.25)	<0.001*
Districts					
Ad Durayhimi	103	63 (61.2)	Reference	Reference	
Al Marawi’ah	72	54 (75.0)	1.91 (0.98–3.70)	1.98 (0.97–4.03)	0.061
Al Mansuriyah	67	59 (88.1)	4.68 (2.03–10.83)	3.29 (1.35–8.01)	0.009*
Bayt Al Faqiah	551	370 (67.2)	1.30 (0.84–2.00)	1.06 (0.67–1.68)	0.801
Socioeconomic status					
High	275	175 (63.6)	Reference	Reference	
Low	205	160 (78.0)	2.03 (1.35–3.07)	1.33 (0.84–2.11)	0.221
Middle	313	211 (67.4)	1.18 (0.84–1.66)	1.03 (0.71–1.49)	0.869

*OR* odds ratio, *AOR* adjusted odds ratio

* Statistically associated with not using LLIN the night prior to the survey

^a^Calculated from households with universal coverage (1LLIN for every two people)

## Discussion

The present survey aimed to assess ownership and use of LLINs in malaria-endemic areas of Al Hudaydah targeted for universal coverage. Despite the ownership of at least one LLIN by 90.6% of the total households surveyed in the present study, only about a quarter of these households had one LLIN for every two members, the target for universal coverage [7]. Therefore, the LLIN coverage in the present survey exceeds that reported by the 2013 Yemen MIS, where 13.0% of households owned at least one LLIN and 1.7% of households had at least one LLIN for every two people in Al Hudaydah [13]. It is noteworthy that the latter MIS was conducted before the mass distribution campaign that targeted Al Hudaydah which might explain the variation between the findings of the two surveys. The inadequate availability of LLINs among these surveyed communities is still evident, where only half of the de facto population had access to LLINs, a finding that could be interpreted by the fact that this survey was conducted 3 years following the mass distribution campaign. This finding is similar to a study in Southwestern Ethiopia where half of the population (51.9%) had access to LLINs indicating that there is still a wide access gap in these malaria endemic settings targeted for universal coverage [22]. A much lower proportion of population with access to LLINs has been reported in Congo, 3 years following a mass campaign [23]. In the latter study, in a 2-month pre-distribution survey, the proportion of households with at least one LLIN for every two people was 4.1% and the population with access to a LLIN was 22.2% [23]. A decrease in coverage and ownership of LLINs for any reason over time has been reported following distribution campaigns with varying rates in a number of post-distribution surveys in other countries [24–27].

In Yemen, replacement campaigns are planned 3 years following the mass campaigns, as recommended by the WHO [7]; however, there are no additional continuous distribution channels currently in place for the provision of nets for additional members following the mass campaigns. Furthermore, there is no monitoring on the durability or loss of the net for replacement which might lead to the reduction in the coverage level [26, 28–30]. The use to access ratio in this study was 0.37. It is noteworthy, that analysis of data from 93 household surveys in 44 countries, assessing ownership, access and use, have reported that a use to access ratio of less than 0.60 is considered poor and reasons for non-use of the available nets should be investigated [20]. Although increased use of ITNs/LLINs among people with access was reported [10, 22, 25, 31, 32], ownership has not been consistently translated to use [33, 34]. The low usage rate of LLINs among people with access could also be attributed to behavioural factors. Kilian et al. [35] reported that multi-channel BCC campaigns influenced the use of LLINs, with a significant increase of LLIN use by vulnerable populations. BCC activities are included as an important strategic component in previous and current Yemen’s NMCES (2014–2018) towards achieving malaria control and elimination. A communication for behavioural impact (COMBI) plan (2009–2012) was developed in 2009, an approach for BCC, particularly aimed to increase use of LLINs [5]. However, BCC activities in Yemen continue to be under key challenges such as ‘weak activities’ with limited allocation of resources that include both financial and staff for implementation [4, 6].

Despite the low access of children under 5 years to the LLINs (13.7%), a higher proportion (42.5%) of those having access actually slept under them during the night preceding the survey. On the other hand, low access and usage rates were observed among pregnant women (16.4 vs. 20%). MIS 2013 reported that 26.2% of children under 5 years and 29.0% of pregnant women slept under LLINs, respectively [13]. As mentioned previously, it should be noted that proportions of children and pregnant women using LLINs in the MIS were calculated from those households with at least one LLIN (not universally covered). In addition, the different sample sizes used in the two surveys could have contributed to such variations. During the present survey, only 10 of 61 pregnant women had access to LLINs. Therefore, it is rather difficult to compare the proportions of using LLINs in the present survey with the 2013 MIS findings [13].

In pursuit of unveiling the barriers to LLIN use in Al Hudaydah, the association of several factors with not using LLIN among individuals with access was assessed. Proportion of population not using a LLIN during the night preceding the survey in Al Mansuriyah was three times higher than those in Ad Durayhimi. Local cultural or behavioural factors might affect LLIN usage as reported in other previous studies elsewhere [35, 36], and such factors need to be further investigated.

Age was significantly associated with LLIN usage, where children under 5 years were the highest of all age groups having slept under LLINs during the night preceding the survey. As documented in literature, this vulnerable age group usually represents the priority household category to use a net [37–41]. Nevertheless, more than half of children under 5 years are still not using LLINs, highlighting the continuous vulnerability of this age group to malaria infection risk in the surveyed areas targeted for universal coverage. Although the proportion of individuals not using LLINs increased with age, with a higher proportion of LLINs non-use among older children aged 5–15 years, there was no significant difference compared to children under 5 years. The latter older age group encompasses children of school-age and studies have reported low use among this age group [40, 41]. In a recent study in Malawi, school-age children (11–15 years) showed significantly lower bed net use as a result of low access to bed nets within the household [42]. In another study in Malawi, the highest malaria prevalence was observed in school-age children (6–15 years), and this highlights the importance of bed net use among this age group as one of the preventive measures against the disease [43]. In the present study, adolescents over 15 years and adults (age groups 16–45 and >45 years) were significantly less likely to use LLINs compared to children under 5 years, which is in agreement with the findings of previous reports conducted in Liberia [44] and Nigeria [45]. However, other studies reported a higher use of bed nets among older age groups [24, 32, 46]. Both the school-age children and adults are usually identified as asymptomatic parasite carriers within malaria-endemic areas and, therefore, contribute to malaria transmission [43].

The poor physical condition of the LLINs inside households, such as the presence of apparent holes, affects their use. In the present study, having three or more damaged nets was significantly associated with a higher risk for non-use of LLINs. This is in line with the findings of several previous studies elsewhere [36, 38, 47, 48], while Kilian et al. [26] reported that the increasing number of net holes was not a determinant factor of decreased use in Nigeria. In the present study, such bad physical condition of the observed LLINs could have been due to their use since 2013. Studies have shown that with increasing years, poor fabric integrity is evident and thus the serviceable life of a LLIN is reduced leading to the decrease of use [28, 47, 49]. The life span of LLINs can vary in different regions, as shown in studies investigating the durability of LLIN. In Rwanda, a study monitoring the durability of LLIN reported a high number of damaged nets; from five to nine out of ten remaining LLINs were damaged, 2 years following distribution campaign [28], while in Nigeria the net serviceable condition varied in the three states surveyed, Nasarawa, Cross River and Zamfara, with ‘*an estimated median net survival of 3.0, 4.5 and 4.7* *years*’, respectively [26]. Therefore, there is a need for regular monitoring of the physical integrity of the distributed LLINs to determine whether the nets are still in serviceable condition during the duration of the recommended 3 years life span and until the targeted year for the replacement campaigns.

The type of house structure was significantly associated with non-use, where living in huts was significantly associated with a higher risk of not using the LLINs compared to living in other more typical houses in the area. Being rectangular in shape, it is possible that there were difficulties in hanging the LLINs in the circle-shaped huts, which may discourage their use. In this context, inadequate space and house structure in the form of huts have been reported as factors affecting the use of bed nets [36, 50].

It should be noted that risk assessment of not using LLINs in the present survey is limited by the small sample size that might affect the study of some variables such the presence of pregnant women inside households.

## Conclusions

The present study shows low LLIN access and use rates among populations targeted for universal coverage in Al Hudaydah, a malaria-endemic area of high transmission. Residents of Al Mansuriyah, the presence of three or more damaged nets, age groups of 16–45 years or older and living in huts were identified as factors significantly associated with not using LLINs. Low access to LLINs necessitates the need for additional LLIN delivery and distribution channels through continuous routine systems with regular monitoring to replace any lost or damaged net for maintaining the universal coverage of household members with LLINs. The identified risk factors of not using LLINs may call for prioritizing the implementation of BCC activities, which include the COMBI strategy to enhance the usage of LLINs in targeted communities.

## Abbreviations

LLINs: long-lasting insecticidal nets
AOR: adjusted odds ratio
CI: 95% confidence interval
BCC: behaviour change communication
WHO: World Health Organization
EMRO: Eastern Mediterranean Regional Office
NMCES: national malaria control and elimination strategy
IRS: indoor residual spraying
ITNs: insecticide-treated nets
RBM: Roll Back Malaria
MERG: monitoring and evaluation reference group
MIS: malaria indicator survey
NMCP: national malaria control programme
SES: socio-economic status
PCA: principal component analysis
OR: odds ratio
COMBI: communication for behavioural impact
